# Cardiovascular safety of 5α-reductase inhibitors in people with benign prostatic hyperplasia and type 2 diabetes: a propensity score-matched analysis

**DOI:** 10.1093/ehjcvp/pvag003

**Published:** 2026-01-17

**Authors:** Haolan Tu, Chengsheng Ju, Stuart J McGurnaghan, Luke A K Blackbourn, Scottish Diabetes Research Network Epidemiology Group, Peter Hanlon, Robert Lindsay, David McAllister, John Petrie, Naveed Sattar, Brian Kennon, Sam Philip, Scott Cunningham, Ewan Pearson, Huan Wang, William Berthon, Luke Blackbourn, Helen Colhoun, Stuart McGurnaghan, Paul McKeigue, Sarah Wild, Ewan Pearson, Li Wei, Ruth Andrew

**Affiliations:** Institute for Neuroscience and Cardiovascular Research, University of Edinburgh, 47 Little France Crescent, Edinburgh EH16 4TJ, UK; School of Pharmacy, University College London, London WC1H 9EU, UK; MRC Unit for Human Genetics, University of Edinburgh, Edinburgh EH4 2XU, UK; MRC Unit for Human Genetics, University of Edinburgh, Edinburgh EH4 2XU, UK; MRC Unit for Human Genetics, University of Edinburgh, Edinburgh EH4 2XU, UK; School of Medicine, University of Dundee, Dundee DD1 9SY, UK; School of Pharmacy, University College London, London WC1H 9EU, UK; Institute for Neuroscience and Cardiovascular Research, University of Edinburgh, 47 Little France Crescent, Edinburgh EH16 4TJ, UK

**Keywords:** Prostatic hyperplasia, Diabetes mellitus, Type 2, Enzyme inhibitors, Cardiovascular diseases

## Abstract

**Aims:**

5α-Reductase inhibitors are prescribed for the treatment of benign prostatic hyperplasia (BPH) and their use is associated with increased risk of incident type 2 diabetes. This study assessed the long-term cardiovascular safety of 5α-reductase inhibitors in comparison with tamsulosin in people with co-existing BPH and type 2 diabetes.

**Methods and results:**

We performed a retrospective, population-based cohort study using Scottish Diabetes Research Network National Diabetes Dataset (SDRN-NDS) and IQVIA Medical Research Data (IMRD-UK). BPH patients ≥40 years with recorded type 2 diabetes mellitus and ≥2 prescriptions of 5α-reductase inhibitors or tamsulosin (2006–2021) were included. After 1:2 variable ratio propensity score matching, cause-specific Cox proportional-hazard models were used to compute the hazard ratio (HR) of incident major adverse cardiovascular events (MACE). A total of 11 969 patients were included in SDRN-NDS and 16 492 in IMRD-UK, with median follow-up durations of 3.8 (IQR: 1.7–6.8) and 4.8 (2.0–8.3) years, respectively. In SDRN-NDS, the HR of MACE in patients receiving 5α-reductase inhibitors relative to tamsulosin was 1.15 (95% CI 1.03–1.30, *P* = 0.007), driven by increased risk of myocardial infarction (MI) (HR 1.20, 1.03–1.40, *P* = 0.022). This was replicated in IMRD-UK, where HR was 1.26 (1.07–1.47, *P* = 0.008) for MACE and 1.33 (1.10–1.60, *P* = 0.005) for MI. We did not observe any increased risks in stroke, cardiovascular death, microvascular complications of diabetes, or faster progression to insulin-based therapies.

**Conclusion:**

Our retrospective data from two large cohorts suggest that the risk of MACE may be increased among patients with type 2 diabetes taking 5α-reductase inhibitors, potentially driven by increased risk of MI. This supports careful monitoring of macrovascular outcomes when prescribing 5α-reductase inhibitors in this population.

## Graphical Abstract

**Graphical Abstract:**
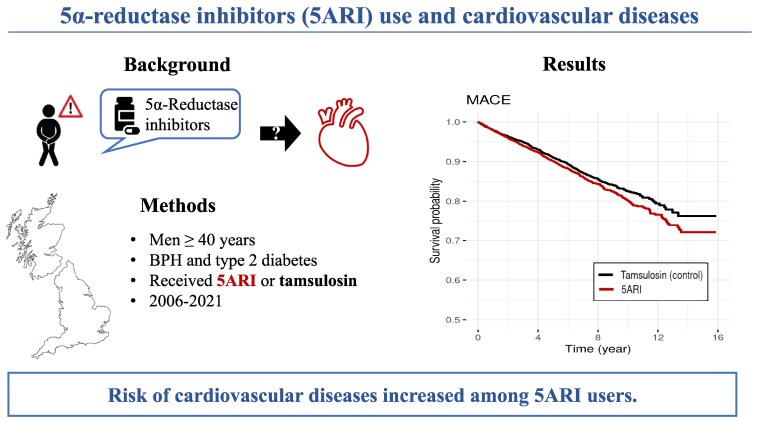

## Introduction

Benign prostate hyperplasia (BPH) affects more than 50% of men over 50 years.^1^ 5α-Reductase inhibitors, finasteride and dutasteride, are widely prescribed to manage the urinary symptoms of BPH.^1^ They work by inhibiting the conversion of testosterone to the more potent dihydrotestosterone (DHT) by the 5α-reductase isozymes and thus reducing androgen-dependent prostate growth.

5α-Reductases are also present in metabolic tissues such as liver, adipose, and skeletal muscles.^2^ Pharmacological inhibition of 5α-reductases in man increases susceptibility to metabolic dysfunction, including insulin resistance, increased HbA_1c_, dyslipidaemia and hepatic lipid accumulation.^2-4^ These effects could be plausibly mediated through suppressed androgen action or modulated metabolism of other enzyme substrates including glucocorticoids. A population-based cohort study has linked both finasteride and dutasteride use to increased risk for new-onset type 2 diabetes.^5^ Similarly, adverse metabolic phenotypes including diet-induced weight gain, impaired glucose tolerance, and increased susceptibility to fatty liver disease were observed in mice lacking 5α-reductase 1 enzyme (*Srd5a1*^-/-^** mice)^6-8^ and following administration of finasteride to obese Zucker rats.^9^

People with type 2 diabetes are at a higher risk of developing cardiovascular diseases^10^ and thus this risk may also be increased in men receiving 5α-reductase inhibitors. The aim of this study was to retrospectively examine cardiovascular effects of finasteride and dutasteride use in patients with co-existing BPH and type 2 diabetes.

## Methods

### Datasets

This study used data from two large UK cohorts: The Scottish Diabetes Research Network—National Diabetes Dataset (SDRN-NDS)^11^ and IQVIA Medical Research Data—UK (IMRD-UK).^12^ SDRN-NDS contains electronic health records of the Scottish Care Information-Diabetes Collaboration (SCI-Diabetes), which captures over 99% of diabetes records in Scotland from 2004 onwards.^11^ The dataset is linked to hospital records in the Scottish Morbidity Records (SMR) and death registry in the National Records of Scotland (NRS). Diagnoses were determined in SDRN-NDS with ICD-10 codes and medical procedures with OPCS-4 codes.

IMRD-UK incorporates data from THIN, a *Cegedim* database, and includes longitudinal GP records of diagnoses, symptoms, biochemistry, and prescription data of approximately 4.5% of the UK population as of 2021.^12^ The data validity and generalizability have been well recorded.^13^ The diagnoses and procedures were recorded using Read Codes.

### Study population

Male individuals were identified from both datasets if they received ≥2 prescriptions of finasteride, dutasteride, or tamsulosin between 1 January 2006 and 30 November 2021. Patients with a diagnosis of type 1 diabetes, alopecia, renal or ureteric calculi, prostate cancer, or any record of prostate surgery were excluded.

For each cohort, eligible patients were divided into three groups: finasteride, dutasteride, and tamsulosin (control). Patients who initiated finasteride or dutasteride, either as monotherapy or in combination with tamsulosin, were assigned to the corresponding 5α-reductase inhibitors group, according to an intention-to-treat analysis framework. Given the small number of patients receiving dutasteride, patients prescribed either finasteride or dutasteride were grouped into a ‘5α-reductase inhibitors (5ARI)’ cohort. The effect of individual drug was evaluated in the subgroup analysis.

For each patient, the second prescription date of finasteride, dutasteride or tamsulosin was defined as the index date. Individuals were eligible if they were ≥40 years and had remained in the dataset for more than 1 year at the index date.

### Clinical characteristics

Baseline covariates extracted in the SDRN-NDS included age; duration of diabetes; social deprivation measured with Scottish index of multiple deprivation;^14^ BMI categories; calendar year at index date; diagnoses of chronic obstructive pulmonary disease (COPD) and cancer (excluding prostate cancer) prior to the index date; mean arterial blood pressure; total cholesterol and non-HDL 2 years prior to the index date; use of ACE inhibitors, angiotensin-receptor blockers, beta blockers, calcium channel blockers, diuretics, statins, oral glucocorticoids, glucose-lowering medications, and nonsteroidal anti-inflammatory drugs (as classified in British National Formulary) 1 year prior to the index date. As self-reported ethnicity data and HbA_1c_ were only available for a subset of patients, they were not included during matching and we considered their balance after matching to reflect residual unmeasured confounding among groups.^15^ Given the low proportion of patients with baseline exposure of DPP-4 inhibitors, SGLT2 inhibitors and GLP-1 receptor agonists, the use of any glucose-lowering medication was included as a binary covariate during matching, and balance of number and type of medications were assessed after matching.

In IMRD-UK, the covariates included age, calendar year, duration of diabetes, number of GP visits in the past year, Townsend deprivation score,^16^ BMI categories, smoking status, and alcohol consumption, previous diagnoses of COPD, cancer, hypertension, and dyslipidaemia, and use of medications as above. HbA_1c_ was not routinely recorded and ethnicity not captured at an individual level within IMRD-UK.

### Study outcomes

In SDRN-NDS, the primary outcome included incident major adverse cardiovascular events (MACE), defined as cardiovascular death, non-fatal myocardial infarction (MI), or non-fatal ischaemic stroke (ICD-10 codes: cardiovascular death, I20–23 and I60–64; MI, I21; and stroke I63). Secondary outcomes included individual components of MACE, peripheral vascular disease (ICD-10 codes: E11.5, I70.2, I73., I74.2–.9, I79.2); nephropathy (the earliest record of 2 consecutive readings of eGFR <30 mL/min/1.73 m^2^ more than 90 days apart, derived from MDRD equations); background (R1–4, M1–2) and referrable (R3–4, M2) diabetic eye diseases; diabetic neuropathy (absence of sensation in monofilament test); and first receipt of insulin-based medications. All secondary outcomes were incident events, and patients with the outcome at baseline were excluded from the corresponding analysis.

As cause of death was not captured in IMRD-UK, incident MACE was defined as first occurrence of fatal and non-fatal MI or ischaemic stroke. Secondary outcomes included individual components of MACE and receipt of insulin-based medications.

All patients were followed up until the occurrence of outcome of interest, death, end of registration, or end of the study period on 30 November 2021, whichever came first.

### Statistical analysis

Propensity score matching was used to adjust for baseline confounding between groups. The propensity score was calculated using logistic regression conditioned on baseline covariates as listed above. The exposed patients were matched without replacement at 1:2 variable ratio to controls within ±0.05 calliper of the propensity score.^17^ Extreme values (BMI <13 kg/m^2^ or >100 kg/m^2^) were truncated. Missing data were grouped as the ‘Missing’ category during matching. Balance of covariates after matching was checked with standardized mean difference (SMD).

Baseline characteristics of each group were summarized with descriptive statistics and difference between groups with SMD. Incidence rates were calculated as the number of newly diagnosed patients per 10 000 person years with 95% Poisson confidence intervals (CIs). Cause-specific Cox proportional hazard models were conducted to calculate the hazard ratio (HR) and 95% CIs between groups. The proportionality assumptions were checked by plotting Schoenfeld residuals. Kaplan–Meier curve and log-rank tests were used to compare the outcomes among groups.

### Sensitivity analyses

A number of sensitivity analyses were performed to ensure the robustness of results. In 2014, the National Institute for Health and Care Excellence (NICE) recommended adding a 5α-reductase inhibitor to α-blockers as the second-line treatment for BPH. Therefore, those patients receiving both 5α-reductase inhibitor and tamsulosin were included in the main analysis, consistent with the changes in clinical practice guidelines. Further, to overcome the potential lead-time bias, a sensitivity analysis was performed using an ‘Only’ cohort where patients were only ever administered finasteride or dutasteride alone, without tamsulosin. Secondly, to account for the significant reduction in admissions and diagnoses of cardiovascular diseases during the Covid-19 lockdown,^18^ the study period was limited from 1 January 2006 to 29 February 2020. A competing risk analysis was also conducted for primary endpoints using the Fine-Gray method with non-cardiovascular death (SDRN-NDS) and all-cause mortality (IMRD-UK) as competing events.

### Subgroup and secondary analyses

The individual effect of finasteride and dutasteride was evaluated in the subgroup analysis and patients co-prescribed finasteride and dutasteride were characterized according to the first prescription. Propensity score matching was performed separately with tamsulosin for finasteride and dutasteride groups. In IMRD-UK, we also performed a secondary analysis in all patients with BPH, where diagnosis of type 2 diabetes was considered as a binary covariate during matching. All analysis was conducted using R (version 4.2.2, R Foundation for Statistical Computing, Vienna, Austria).

## Results

### Baseline characteristics

**Figure 1:**
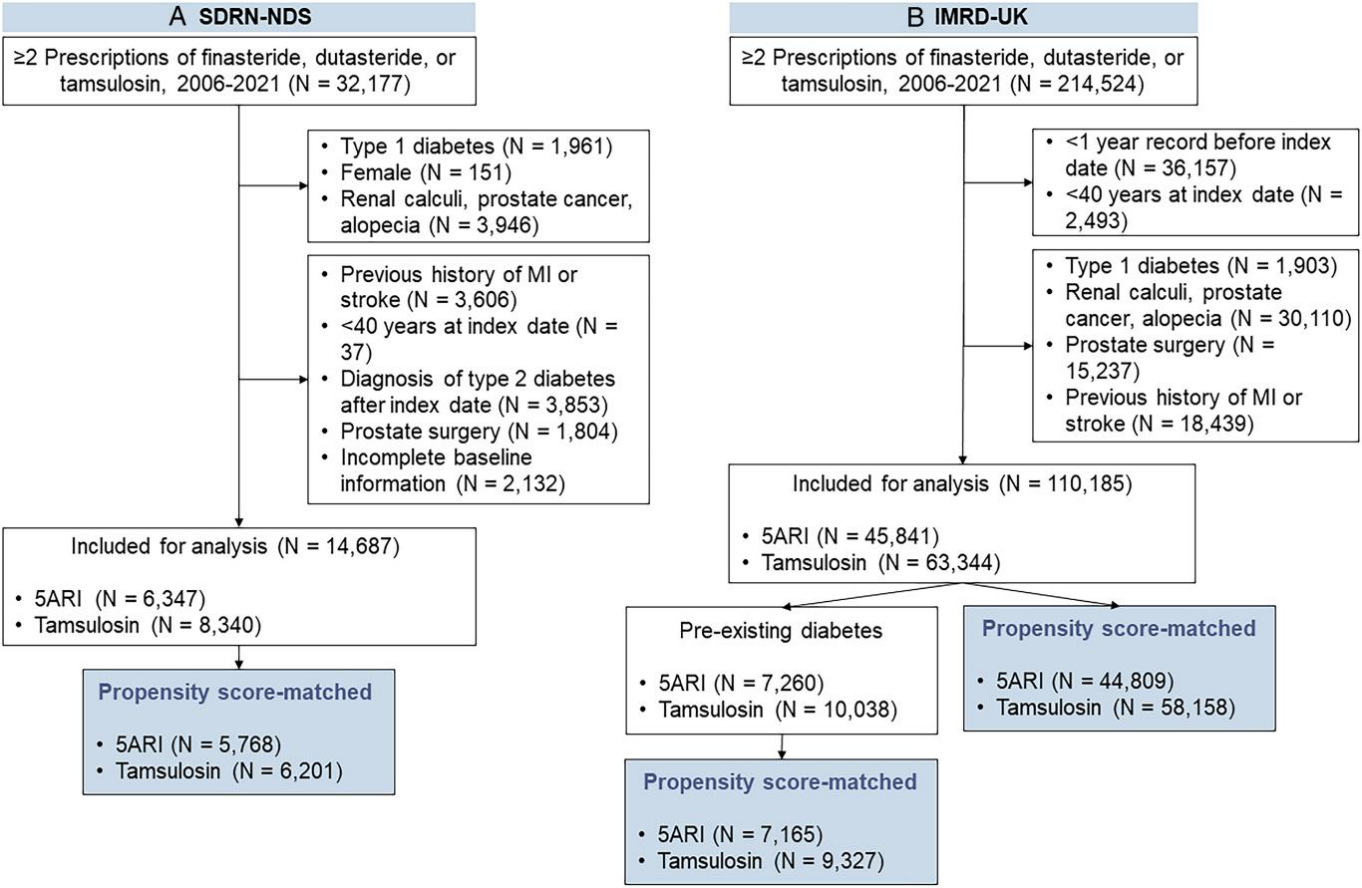
Consort diagram illustrating the study selection process in (*A*) SDRN-NDS and (*B*) diabetic cohort in the IMRD-UK. Abbreviation: 5ARI, 5α-reductase inhibitors.

Within SDRN-NDS, 36 415 patients received more than one prescription of finasteride, dutasteride or tamsulosin between January 2006 and November 2021, and 14 687 fulfilled the inclusion criteria (see Supplementary material online, *Table S1*). A total of 11 969 patients were included after propensity score matching, which included 5768 patients in the 5ARI group and 6201 tamsulosin (*Figure 1*). Their baseline characteristics were included in *Table 1*. Variables were balanced after matching except baseline age between 5ARI (72.4 years) and tamsulosin (71.0 years) users (SMD = 0.150), which was caused by variable ratio matching. We assessed the baseline characteristics with the first-level matches and covariates were well-balanced (see Supplementary material online, *Table S2*).

**Table 1:** Baseline characteristics of 5α-reductase inhibitors and tamsulosin groups in the propensity score-matched SDRN-NDS cohort

	Tamsulosin	5ARI	SMD
***N***	6201	5768	
Age	71.0 (9.4)	72.4 (8.8)	0.150
Diabetes duration (year)	10.1 (7.5)	10.2 (7.5)	0.022
SIMD quintile			0.044
1 (most deprived)	997 (16.1)	981 (17.0)	
2	1357 (21.9)	1200 (20.8)	
3	1318 (21.3)	1258 (21.8)	
4	1293 (20.9)	1158 (20.1)	
5 (least deprived)	994 (16.0)	960 (16.6)	
Missing	242 (3.9)	211 (3.7)	
BMI categories (kg/m^2^)			0.047
<25	848 (13.7)	842 (14.6)	
25–30	2247 (36.2)	2165 (37.5)	
>30	2967 (47.8)	2629 (45.6)	
Missing	139 (2.2)	132 (2.3)	
Smoking			0.038
No	2269 (36.6)	2077 (36.0)	
Former	2949 (47.6)	2839 (49.2)	
Yes	768 (12.4)	664 (11.5)	
Missing	215 (3.5)	188 (3.3)	
Calendar year			0.121
2006	141 (2.3)	128 (2.2)	
2007	123 (2.0)	177 (3.1)	
2008	184 (3.0)	176 (3.1)	
2009	213 (3.4)	226 (3.9)	
2010	348 (5.6)	281 (4.9)	
2011	326 (5.3)	352 (6.1)	
2012	403 (6.5)	408 (7.1)	
2013	428 (6.9)	359 (6.2)	
2014	464 (7.5)	405 (7.0)	
2015	459 (7.4)	430 (7.5)	
2016	525 (8.5)	417 (7.2)	
2017	480 (7.7)	449 (7.8)	
2018	553 (8.9)	561 (9.7)	
2019	504 (8.1)	491 (8.5)	
2020	477 (7.7)	460 (8.0)	
2021	573 (9.2)	448 (7.8)	
COPD	455 (7.3)	474 (8.2)	0.033
Cancer	713 (11.5)	675 (11.7)	0.006
Biochemistry			
Total cholesterol (mmol/L)	4.1 (0.9)	4.1 (0.9)	0.038
Non-HDL (mmol/L)	3.0 (0.9)	2.9 (0.9)	0.045
Mean arterial pressure (mm Hg)	95.6 (8.0)	95.2 (8.0)	0.051
HbA_1c_ (mmol/mol)	58.8 (17.1)	56.8 (15.5)	0.121
Medications			
ACE inhibitors	3067 (49.5)	2852 (49.4)	<0.001
ARB	1111 (17.9)	1041 (18.0)	0.003
Beta-blockers	2103 (33.9)	1987 (34.4)	0.011
Calcium channel blockers	2303 (37.1)	2174 (37.7)	0.011
Diuretics	2124 (34.3)	2042 (35.4)	0.024
Statins	4876 (78.6)	4552 (78.9)	0.007
Oral glucocorticoids	879 (14.2)	834 (14.5)	0.004
NSAID	1765 (28.5)	1657 (28.7)	0.006
Glucose-lowering medications	4587 (74.0)	4211 (73.0)	0.022
Metformin	3888 (62.7)	3481 (60.4)	0.048
Sulfonylurea	1997 (32.2)	1771 (30.7)	0.032
DPP4 inhibitor	668 (10.8)	589 (10.2)	0.018
SGLT2 inhibitor	362 (5.8)	251 (4.4)	0.068
GLP-1 RA	195 (3.1)	154 (2.7)	0.028
Number of glucose-lowering medications			0.061
0	1614 (26.0)	1557 (27.0)	
1	2038 (32.9)	1977 (34.3)	
2	1438 (23.2)	1340 (23.2)	
3	661 (10.7)	577 (10.0)	
3+	450 (7.3)	317 (5.3)	
Number of drugs	1.43 (1.25)	1.35 (1.20)	0.079

Data represented in *N* (%) or mean (SD). Standardized mean difference (SMD) calculated with reference to tamsulosin group.

Abbreviations: 5ARI, 5α-reductase inhibitors; SIMD, Scottish index of multiple deprivation; COPD, chronic obstructive pulmonary disease; ARB, angiotensin II receptor blocker; NSAID, non-steroidal anti-inflammatory drugs; DPP4, dipeptidyl peptidase-4; SGLT2, sodium-glucose co-transporter-2 inhibitors; GLP-1 RA, glucagon-like peptide-1 (GLP-1) receptor agonists.

In IMRD-UK, 17 298 patients fulfilled the selection criteria (see Supplementary material online, *Table S3*). The matched cohort included 16 492 patients, of which 7165 were in the 5ARI group and 9327 tamsulosin. Similarly, variables were balanced after matching (*Table 2*).

**Table 2:** Baseline characteristics of 5α-reductase inhibitors and tamsulosin groups in the propensity score-matched diabetic cohort in IMRD-UK

	Tamsulosin	5ARI	SMD
***N***	9327	7165	
Age	71.4 (9.1)	73.4 (8.7)	0.215
GP visits in past year	14.2 (10.4)	14.9 (10.3)	0.065
Diabetes duration (year)	8.8 (6.7)	9.0 (6.9)	0.035
Townsend			0.034
1 (least deprived)	1842 (19.7)	1374 (19.2)	
2	1799 (19.3)	1466 (20.5)	
3	1777 (19.1)	1335 (18.6)	
4	1522 (16.3)	1163 (16.2)	
5 (most deprived)	1052 (11.3)	780 (10.9)	
Missing	1335 (14.3)	1047 (14.6)	
BMI category (kg/m^2^)			0.043
<25	1529 (16.4)	1237 (17.3)	
25–30	3581 (38.4)	2840 (39.6)	
>30	3975 (42.6)	2911 (40.6)	
Missing	242 (2.6)	177 (2.5)	
Smoking			0.043
**���**No	3376 (36.2)	2618 (36.5)	
Former	4938 (52.9)	3861 (53.9)	
Yes	1007 (10.8)	681 (9.5)	
Missing	6 (0.1)	5 (0.1)	
Alcohol consumption			0.015
No	814 (8.7)	610 (8.5)	
Former	1524 (16.3)	1205 (16.8)	
Yes	6705 (71.9)	5140 (71.7)	
Missing	284 (3.0)	210 (2.9)	
Calendar year			0.079
2006	336 (3.6)	299 (4.2)	
2007	362 (3.9)	305 (4.3)	
2008	430 (4.6)	374 (5.2)	
2009	481 (5.2)	417 (5.8)	
2010	657 (7.0)	492 (6.9)	
2011	681 (7.3)	568 (7.9)	
2012	732 (7.8)	578 (8.1)	
2013	794 (8.5)	625 (8.7)	
2014	799 (8.6)	569 (7.9)	
2015	770 (8.3)	545 (7.6)	
2016	659 (7.1)	462 (6.4)	
2017	584 (6.3)	463 (6.5)	
2018	643 (6.9)	451 (6.3)	
2019	591 (6.3)	426 (5.9)	
2020	429 (4.6)	330 (4.6)	
2021	379 (4.1)	261 (3.6)	
COPD	1038 (11.1)	821 (11.5)	0.010
Hypertension	6124 (65.7)	4843 (67.6)	0.041
Dyslipidaemia	2107 (22.6)	1618 (22.6)	<0.001
Cancer	2148 (23.0)	1764 (24.6)	0.037
Baseline medications			
ACE inhibitors	4352 (46.7)	3308 (46.2)	0.010
ARB	1617 (17.3)	1278 (17.8)	0.013
Beta-blockers	2517 (27.0)	2034 (28.4)	0.031
Calcium channel blockers	3260 (35.0)	2517 (35.1)	0.004
Diuretics	2474 (26.5)	2065 (28.8)	0.051
Statins	6916 (74.2)	5325 (74.3)	0.004
Oral glucocorticoids	511 (5.5)	392 (5.5)	<0.001
NSAID	812 (8.7)	558 (7.8)	0.033
Glucose-lowering medications	7116 (76.3)	5304 (74.0)	0.053
Metformin	6347 (68.0)	4602 (64.2)	0.081
Sulfonylurea	2986 (32.0)	2184 (30.5)	0.033
DPP4 inhibitor	1143 (12.3)	827 (11.5)	0.022
SGLT2 inhibitor	332 (3.6)	204 (2.8)	0.040
GLP-1 RA	270 (2.9)	172 (2.4)	0.031
Number of glucose-lowering medications			0.078
0	2065 (22.1)	1750 (24.4)	
1	3585 (38.4)	2836 (39.6)	
2	2556 (27.4)	1805 (25.2)	
3	901 (9.7)	604 (8.4)	
3+	220 (2.4)	170 (2.4)	
Number of drugs	1.32 (1.01)	1.25 (1.00)	0.069

Data represented in *N* (%) or mean (SD). Standardized mean difference (SMD) calculated with reference to tamsulosin group.

Abbreviations: 5ARI, 5α-reductase inhibitors; GP, general practice; COPD, chronic obstructive pulmonary disease; ARB, angiotensin II receptor blocker; NSAID, non-steroidal anti-inflammatory drugs; DPP4, dipeptidyl peptidase-4; SGLT2, sodium-glucose co-transporter-2 inhibitors; GLP-1 RA, glucagon-like peptide-1 (GLP-1) receptor agonists.

### Use of 5ARI and cardiovascular events

**Figure 2:**
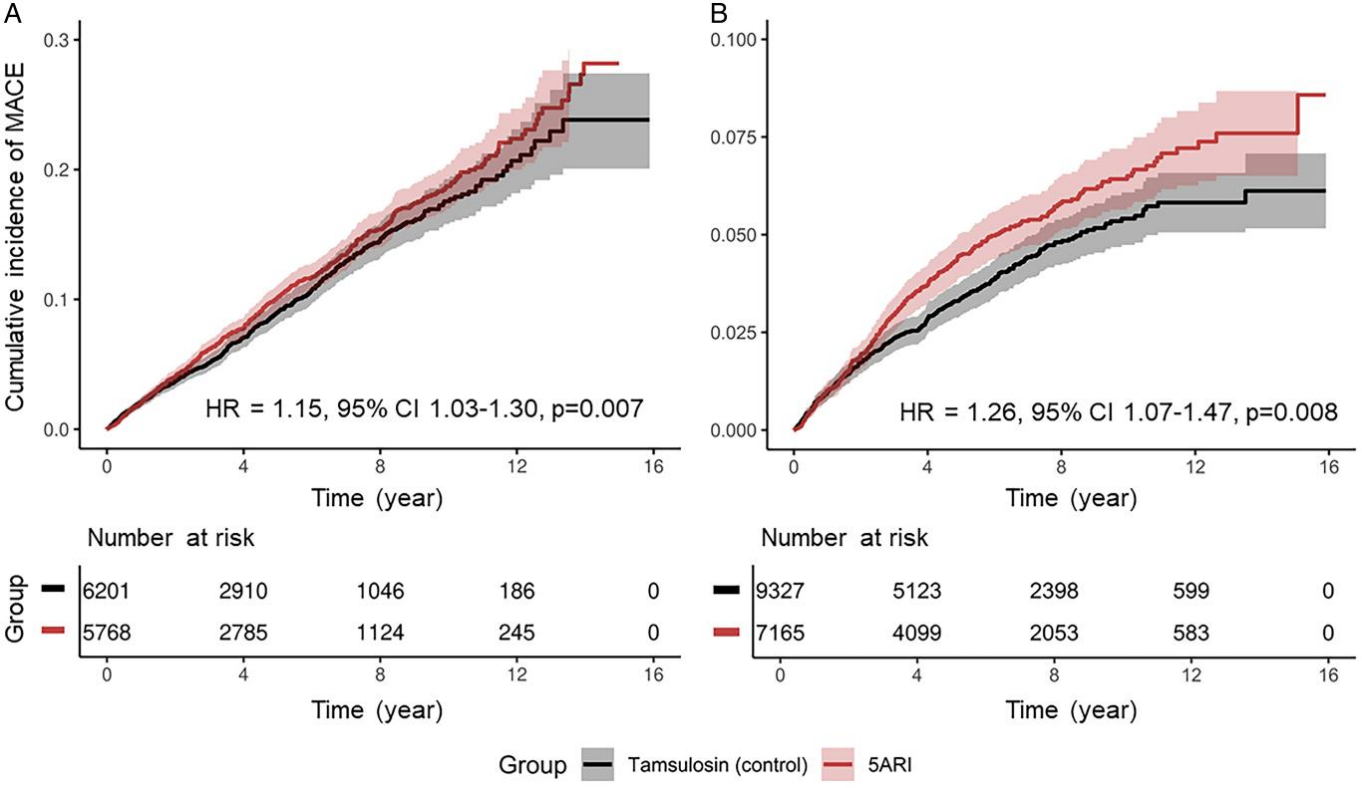
Cumulative incidence plot of major adverse cardiovascular events (MACE) in the propensity score-matched (*A*) SDRN-NDS and (*B*) diabetic cohort in IMRD-UK. Abbreviation: 5ARI, 5α-reductase inhibitors.

In SDRN-NDS, a total of 1099 episodes of MACE were recorded over a median follow-up of 3.8 (IQR: 1.7–6.8) years, of which there were 573 in the 5ARI group and 526 in tamsulosin (see Supplementary material online, *Table S4*). The incidence rate for MACE was 212.0 per 10 000 person years (95% CI: 194.7–229.4) for the 5ARI group, and 191.8 (175.4–208.2) for tamsulosin. Similarly, in IMRD-UK, the incidence rate for MACE was higher for the 5ARI group (75.4 per 10 000 person-years, 95% CI: 66.9–83.9) when compared with tamsulosin (61.7, 95% CI: 54.8–68.6). *Figure 2* shows the increased probability of incident MACE among patients in the 5ARI group in both cohorts. This increase was also observed in all individual components of MACE in both datasets. The cumulative incidence plots of all individual components of MACE are shown in Supplementary material online, *Figures S1* and *S2*.

**Figure 3:**
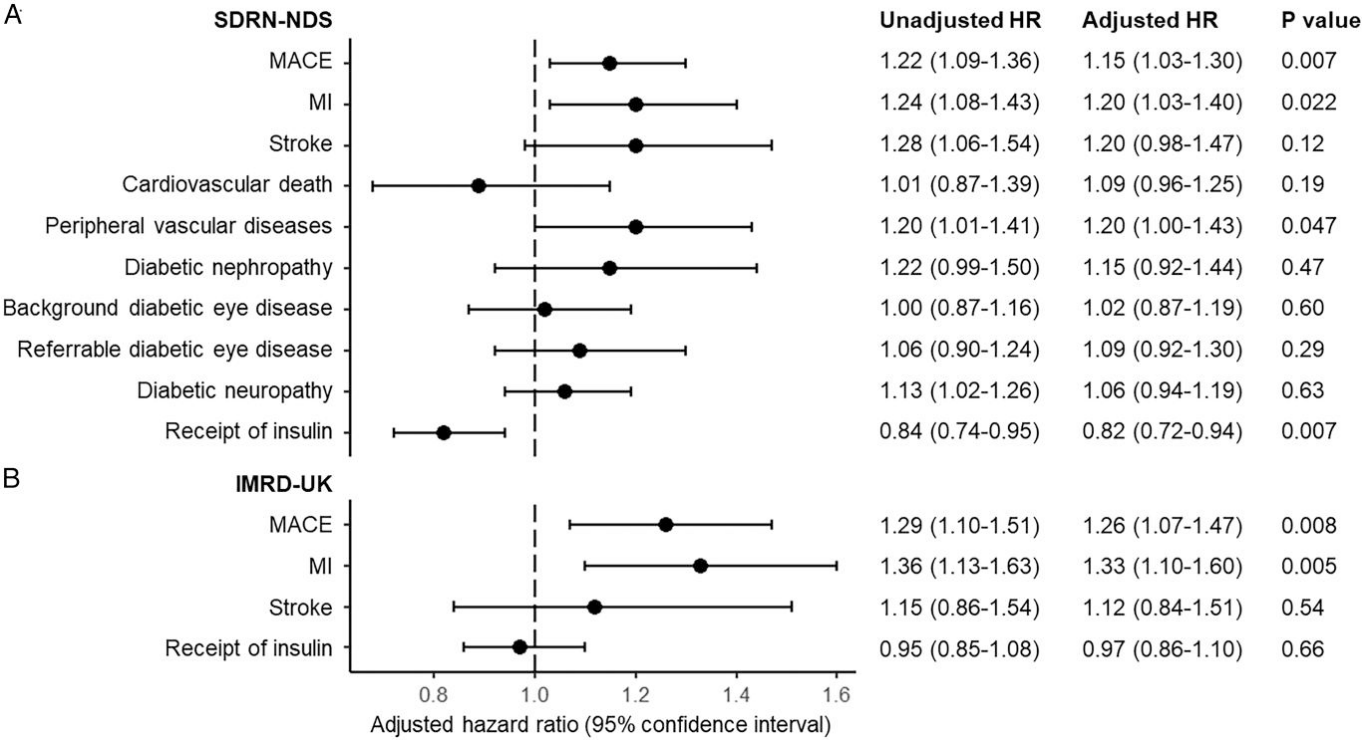
Hazard ratio (HR) and 95% confidence interval for all outcomes in (*A*) SDRN-NDS and (*B*) diabetic cohort in IMRD-UK. Major adverse cardiovascular events (MACE) were defined as non-fatal myocardial infarction (MI), non-fatal stroke, or cardiovascular death in SDRN-NDS, and fatal or non-fatal MI and stroke in IMRD-UK.

*Figure 3* shows the results of all estimated Cox proportional hazard models. In SDRN-NDS, there was an increased risk of MACE, MI, and stroke but not cardiovascular death among users of 5α-reductase inhibitors before propensity score matching. In the matched cohort, the HR of MACE is 1.15 (95% CI 1.03–1.30, *P* = 0.007) for 5ARI when compared with tamsulosin. An increase in risk was observed for incidence MI (HR 1.20, 95% CI 1.03–1.40, *P* = 0.022) but not stroke (HR 1.20, 95% CI 0.98–1.47, *P* = 0.12) or cardiovascular death (HR 0.89, 95% CI 0.68–1.15, *P* = 0.19). Among secondary endpoints, the HR for peripheral vascular diseases was 1.20 (95% CI 1.00–1.43, *P* = 0.047). We did not observe increase in risk for any other microvascular endpoints including diabetic nephropathy, background or referrable eye diseases, or neuropathy. These findings were replicated in IMRD-UK, where there was an increased risk for incident MACE in the 5ARI group (HR 1.26, 95% CI 1.07–1.47, *P* = 0.008). This was again driven by MI where the HR is 1.33 (95% CI 1.10–1.60, *P* = 0.005). The HR for stroke was 1.12 (95% CI 0.84–1.51, *P* = 0.54). A small decrease in risk for time-to-receipt of insulin-based therapy was found in SDRN-NDS (HR 0.82, 95% CI 0.72–0.94, *P* = 0.007). However, this was not replicated in IMRD-UK, where the HR was 0.97 (95% CI 0.86–1.10, *P* = 0.66).

The results of the sensitivity analyses testing for effect of Covid-19, the ‘Only’ group, and the competing risk analysis are shown in Supplementary material online, *Tables S5*–S7. The analyses were consistent with the primary analyses except the ‘Only’ group in IMRD-UK which were underpowered due to the low sample size (see Supplementary material online, *Tables S8*).

### Use of finasteride, dutasteride, and cardiovascular events

The individual effect of finasteride or dutasteride was evaluated in subgroup analyses. For finasteride, a total of 11 891 patients (5730 in the finasteride group and 6161 matched tamsulosin) were included in SDRN-NDS and 14 703 (5763 finasteride and 8940 matched tamsulosin) in IMRD-UK, respectively. In SDRN-NDS, the HR of finasteride group was 1.14 (1.01–1.28, *P* = 0.04) for incident MACE, 1.19 (1.02–1.39, *P* = 0.03) for non-fatal MI, 1.23 (1.01–1.51, *P* = 0.04) for non-fatal stroke, and 0.93 (0.71–1.21, *P* = 0.64) for cardiovascular death. An increase in risk was found for incident peripheral vascular disease (HR 1.25, 1.04–1.49, *P* = 0.01) but not any other microvascular complications. In IMRD-UK, the HR was 1.14 (0.97–1.33, *P* = 0.11) for MACE, 1.21 (1.00–1.45, *P* = 0.05) for MI, and 1.00 (0.74–1.34, *P* = 0.89) for stroke. We did not observe any increase in risk for faster time to receipt of insulin-based therapy in either dataset (see Supplementary material online, *Figure S3*).

The number of patients prescribed dutasteride was low in both datasets, with 2155 patients in SDRN-NDS (724 dutasteride and 1431 matched tamsulosin) and 4455 in IMRD-UK (1 488 dutasteride and 2967 matched tamsulosin). The HR was 0.90 (0.71–1.16, *P* = 0.72) for MACE in SDRN-NDS, with statistically insignificant results for all individual components. While in IMRD-UK, the risk increase in the dutasteride group was higher than finasteride, when compared with tamsulosin. Here the HR was 1.35 (1.02–1.79, *P* = 0.01) for MACE, 1.48 (1.07–2.05, *P* = 0.01) for MI, and 1.08 (0.64–1.82, *P* = 0.98) for stroke (see Supplementary material online, *Figure S4*). Similar to the main analysis, a small decrease in risk for receipt of insulin was found in SDRN-NDS (HR 0.66, 0.48–0.90, *P* = 0.01), and this was not replicated in the IMRD-UK (HR 1.07, 0.85–1.35, *P* = 0.88).

### Secondary analysis

In IMRD-UK, a secondary analysis was performed in all patients prescribed 5ARIs or tamsulosin for BPH between 2006 and 2021, regardless of their diabetes status at baseline. A total of 110 185 patients fulfilled the inclusion criteria and 102 967 were included after matching over a median follow-up of 5.7 (IQR: 2.4–9.3) years. This included 44 809 patients in the 5ARI group and 58 158 in the matched tamsulosin group (see Supplementary material online, *Table S9*). Pre-existing type 2 diabetes was included as an additional covariate during matching.

**Figure 4:**
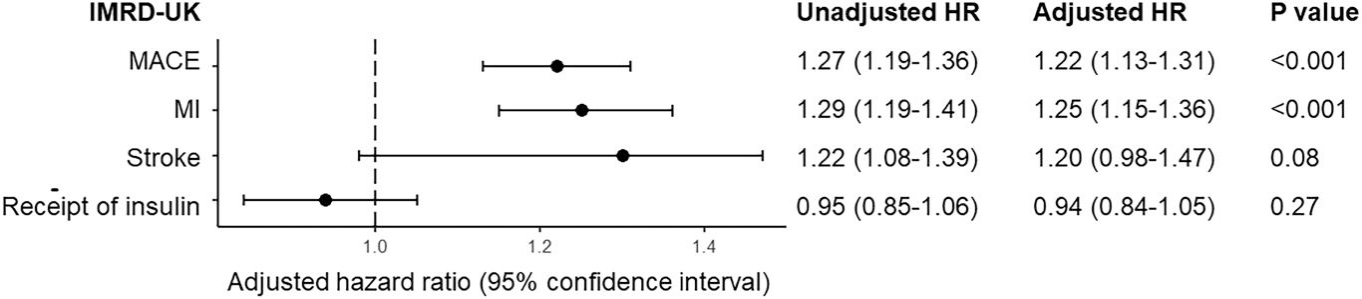
Hazard ratio (HR) and 95% confidence interval for all outcomes in general BPH population in the IMRD-UK. Major adverse cardiovascular events (MACE) were defined as fatal or non-fatal MI and stroke in IMRD-UK.

The results were consistent with the primary analysis (*Figure 4*; Supplementary material online, *Table S10*). The increased risk in MACE was present for the 5ARI group (HR 1.22, 1.13–1.31, *P* < 0.001) and separately for finasteride (HR 1.17, 1.09–1.26, *P* < 0.001), and dutasteride (HR 1.17, 1.02–1.33, *P* = 0.006). This was again mainly driven by MI, where the HR was 1.25 (1.15–1.36, *P* < 0.001) for 5ARI group, 1.19 (1.10–1.30, *P* < 0.001) for finasteride, and 1.23 (1.06–1.43, *P* = 0.02) for dutasteride.

## Discussion

In this study, we built upon our previous work demonstrating the increased risk of incident type 2 diabetes following use of 5α-reductase inhibitors,^5^ against the setting that patients with type 2 diabetes are highly susceptible to cardiovascular complications. We examined cardiovascular outcomes of 5α-reductase inhibitors in SDRN-NDS, a dataset with populational coverage of people living with diabetes in Scotland, and IMRD-UK, where patients with type 2 diabetes were followed in a primary care setting. In both datasets, we observed an increased risk of developing incident MACE among users of 5α-reductase inhibitors, primarily driven by increased risk of incident myocardial infarction, when controlling for age and other confounding factors. This increased risk was also present in all patients prescribed 5α-reductase inhibitors for BPH, regardless of their diabetes status at drug initiation. We did not observe increased risk of ischaemic stroke, cardiovascular death, microvascular complications of diabetes, or reduction in time to receipt of insulin-based therapy. These findings underscore the importance of monitoring the long-term cardiovascular safety profile in individuals prescribed those medications.

In this study, we used an active comparator design with BPH patients prescribed tamsulosin as controls. Several α-blockers are licensed in the UK for the management of lower urinary tract symptoms associated with BPH, including alfuzosin, doxazosin, prazosin, tamsulosin, and terazosin. They work by antagonizing α_1_-adrenergic receptors on smooth muscle cells, where the α_1A_ receptor subtype is the most predominant subtype in human prostate, bladder neck, and urethra.^19^ Tamsulosin was selected in our study as it is the most widely prescribed uroselective α-blocker in BPH, while non-selective α-blockers (doxazosin, prazosin and terazosin) are also prescribed for hypertension.^20^  ^,21^ Tamsulosin displayed high affinity for the α_1A_ receptor subtype, resulting in its greater uroselectively and therefore less potential for adverse cardiovascular effects.^22,23^ Propensity score matching was used to balance the demographic and clinical covariates between patients in the 5ARI and tamsulosin groups. Baseline HbA_1c_ and the type of glucose-lowering medications were not included in the matching process, though they were balanced in the matched cohort.

While BPH remains the most common indication for 5α-reductase inhibitors prescriptions, both drugs are sometimes used in the treatment of alopecia and also for prostate cancer and hirsutism (albeit unlicensed in the UK). Thus, patients with any record of those indications other than BPH were excluded to maintain a homogenous study population. Similarly, we excluded patients with renal or ureteric calculi where use of tamsulosin was indicated recently.^24^ We also excluded patients who had undergone surgical intervention for BPH, as they would not require further symptom-control medications after the surgery. In both IMRD-UK and SDRN-NDS, we observed an interesting temporal trend in the use of dutasteride, where prescription numbers have been decreasing since early 2010s (see Supplementary material online, *Figure S5*). This might be explained by finasteride coming off label around that time. To account for these changes in clinical practice, we used calendar year as a covariate in the multivariable model. Due to numbers of dutasteride users being low in some settings, we grouped both finasteride and dutasteride into a ‘5ARI group’ to investigate the effect of drug class.

There are a few limitations to consider. First of all, as of all retrospective population-based studies, bias may arise from the accuracy and completeness of available diagnosis and prescriptions records. There was a notable difference between incidence of cardiovascular diseases between the Scottish and English cohorts (incidence of MI in 5ARI users: 122.9 and 57.4 per 10 000 person years, respectively), despite very similar baseline characteristics. This is generally consistent with incidence rates found in previous studies.^25,26^ The two datasets were collected in different care settings and thus status was recorded using different coding systems. Diagnoses were obtained from hospital in-patient records in SDRN-NDS using ICD-10 codes and from primary care data in IMRD-UK using Read codes. While the validity and generalizability of both datasets in diabetes and cardiovascular research have been well recorded,^11,13,25^ certain macrovascular and microvascular complications of diabetes are managed in secondary care and may not be robustly captured using primary care Read codes alone. For this reason, we studied specific parameters only in the SDRN-NDS, which integrates detailed laboratory tests relevant to diabetes management, diabetic foot screening outcomes, and retinopathy grading from the SCI-Diabetes.^11^ Lastly, adherence was not measured as the medication record was identified from primary care records, and the results could only be interpreted under an intention-to-treat framework. However, we limited the study cohort to patients prescribed more than one prescription of the study drugs, who were more likely to be long-term users. While our study provided insights through real-world data, a dedicated prospective study or randomized controlled trial with longer follow-up would be warranted to validate our observations and address the potential limitations.

Previous studies on the cardiovascular safety of 5α-reductase inhibitors have yielded mixed results. The REDUCE trial found that dutasteride use was associated with increased risk of composite cardiac failure when compared with placebo,^27^ although a meta-analysis of 12 randomized controlled trials did not find any increased risks of adverse cardiovascular diseases.^28^ In contrast, population-based cohort studies have reported either no significant difference in hospitalization for heart failure^29^ or lowered risk for incident cardiac failure^30^ compared with tamsulosin, though these studies were indexed at time of BPH diagnosis and thus subjected to prevalent user bias and immortal time bias.^31^ Similarly, a cohort study in Korean men did not report a significant risk difference in cardiovascular diseases and but instead reduced risk in patients with highest exposure to 5α-reductase inhibitors,^32^ with drug exposure assessed during the period before the start of follow-up. A previous study also reported reduction in the 1-year risk of MACE in comparison with tamsulosin although it should be recognized this follow-up period was short.^33^ A nation-wide cohort study in Taiwan reported a lower cumulative rate of cardiovascular diseases in users of 5α-reductase inhibitors compared with tamsulosin.^34^ However, it did not adjust for cardiometabolic risk factors such as BMI. It is important to note that these studies did not specifically focus on people with diabetes, who might be more susceptible to the long-term cardiovascular consequences of finasteride or dutasteride.

The disparity between the link between pharmacological inhibition of 5α-reductases and increased risk of myocardial infarction but not ischaemic stroke or microvascular endpoints was unexpected. While the mechanism underlying this selective risk difference is unclear, it might be attributed to the distinct aetiologies between MI and stroke. Most acute MIs are caused by atherosclerotic plaque rupture or erosion, while causes of acute ischaemic stroke are more heterogenous.^35-37^ There is a strong link between adverse plasma lipid profiles and MI,^38,39^ and long-term dutasteride has been shown to increase serum total cholesterol and LDL levels.^4^ Further research is needed to investigate whether pharmacological inhibition of 5α-reductase activities may selectively influence pathways that are more relevant to MI.

A number of mechanisms can be proposed to explain the increased cardiometabolic risks observed. 5α-Reductases facilitate the A-ring reduction of 3-keto, Δ^4,5^ C19/C21 steroids, including cortisol, androgens, progestogens, and mineralocorticoids, all of which have well recognized effects on energy or vascular homeostasis.^40-43^ Alterations in 5α-reductase activities may contribute to cardiometabolic dysfunction via dysregulation of steroid actions, most plausibly through a state of glucocorticoids excess or androgen deprivation. Glucocorticoids regulate fuel metabolism and increase the risk of cardiovascular diseases when in excess.^44^ 5α-Reductase deficiency in mice increases accumulation of glucocorticoids within metabolic tissues such as liver and adipose,^45^ although the propensity to alter risk of plaque development has not been assessed, for example, by crossing with *ApoE*-deficient mice. Moreover, 5α-reduced glucocorticoids retain anti-inflammatory actions, thus depletion of these steroids may promote vascular inflammation.^46^ Similarly, 5α-reductases converts testosterone to the more potent androgen DHT and pharmacological inhibition of the enzyme activities contribute to low circulatory DHT.^2^ The adverse metabolic and vascular outcomes of androgen deficiency in men are well-recorded as seen in men with hypogonadism.^47,48^ In rodents, finasteride induced hepatic steatosis in both castrated and intact mice, suggesting this adverse metabolic effect might be primarily due to glucocorticoids rather than androgens.^9^ Increased activity of the hypothalamus-pituitary-adrenal (HPA) axis and impaired negative feedback of cortisol have been reported in people with type 2 diabetes.^49,50,51^ Similarly, low serum testosterone levels are also prevalent among men with type 2 diabetes.^52,53^ To our knowledge, there is no direct study available measuring 5α-reductase activity in people with type 2 diabetes, although increased 5α-reduction of cortisol has been reported indirectly through urinary metabolite profiling of people with type 2 diabetes.^54^ Future study is warranted to investigate whether synergistic interactions occur between hypogonadism and 5α-reductase inhibition, or between hypercortisolism and 5α-reductase inhibition (e.g. during co-prescription of synthetic glucocorticoids and 5α-reductase inhibitors).

5α-Reductase inhibitors are also prescribed off-label in women for treatment of polycystic ovarian syndrome and hirsutism. However, women were not included in this study due to the low number of patients. Effects of 5α-reductase inhibitors in women merits further research as female mice lacking 5α-reductase 1 enzyme (*Srd5a1^-/-^*) were more susceptible to hyperinsulinemia on high fat diet than their male counterparts,^6^ suggesting a potential sexual dimorphism in the magnitude of response to the drug treatment.

## Conclusion

We have demonstrated that the risk of MACE is increased in BPH patients prescribed 5α-reductase inhibitors through a propensity-score matched analysis of two UK-based cohorts. This is driven by an increased risk of incident MI, but not other cardiovascular outcomes including stroke, cardiovascular death, or microvascular complications of diabetes. Adverse macrovascular events should be considered as a potential longer term side effect of 5α-reductase inhibitors use, especially in patients with existing type 2 diabetes.

## Supplementary Material

pvag003_Supplementary_Data

## Data Availability

The data supporting the findings of this study are derived from the SDRN-NDS and IMRD-UK. Approvals for data linkage and use of data for research purposes for SDRN-NDS were obtained from the Public Benefit and Privacy Panel for Health and Social Scare (HSC-PBPP; Reference: 1617-0147) and the West of Scotland Research Ethics Committee (Reference: 21/WS/0047). Access to SDRN-NDS data is restricted and available upon reasonable request through the Scottish Diabetes Research Network. IMRD-UK data are provided by patients as part of routine primary care and are available upon application to IQVIA’s Scientific Research Committee (SRC Reference Number: 21SRC006).
