# Supplementary material for: Cardiovascular safety of 5α-reductase inhibitors in people with benign prostatic hyperplasia and type 2 diabetes: a propensity score-matched analysis
Source: Eur Heart J Cardiovasc Pharmacother. 2026 Jan 17;12(2):97–107. doi: 10.1093/ehjcvp/pvag003 (PMC12946974; doi:10.1093/ehjcvp/pvag003)
Supplement: pvag003_Supplementary_Data [file pvag003_supplementary_data.zip › SDRN epi group members Oct 2025.docx]

**Scottish Diabetes Research Network epidemiology group members – October 2025**

University of Glasgow, Glasgow, UK

Peter Hanlon

Robert Lindsay

David McAllister

John Petrie

Naveed Sattar

Queen Elizabeth Hospital, Glasgow, UK

Brian Kennon

Grampian Diabetes Research Unit, Aberdeen, UK

Sam Philip

University of Dundee, Dundee, UK,

Scott Cunningham

Ewan Pearson

Huan Wang

University of Edinburgh, Edinburgh, UK

William Berthon

Luke Blackbourn

Helen Colhoun

Stuart McGurnaghan

Paul McKeigue

Sarah Wild
