# Supplementary material for: Cardiovascular safety of 5α-reductase inhibitors in people with benign prostatic hyperplasia and type 2 diabetes: a propensity score-matched analysis
Source: Eur Heart J Cardiovasc Pharmacother. 2026 Jan 17;12(2):97–107. doi: 10.1093/ehjcvp/pvag003 (PMC12946974; doi:10.1093/ehjcvp/pvag003)
Supplement: pvag003_Supplementary_Data [file pvag003_supplementary_data.zip › Tu2026.ehjcvp.supp_figures.pdf]

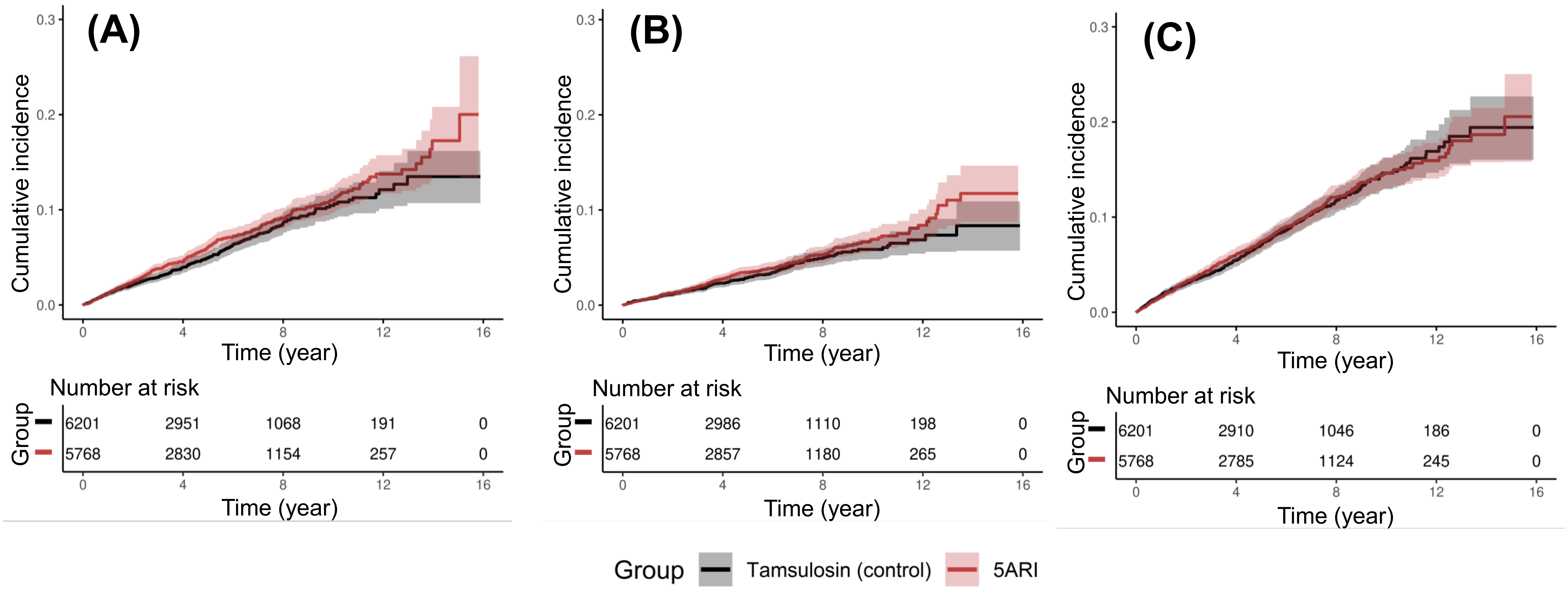

Tu et al. Supplementary Figure 1: Cumulative incidence plot of (A) myocardial infarction, (B) stroke, and (C) cardiovascular death in the matched SDRN-NDS cohort.

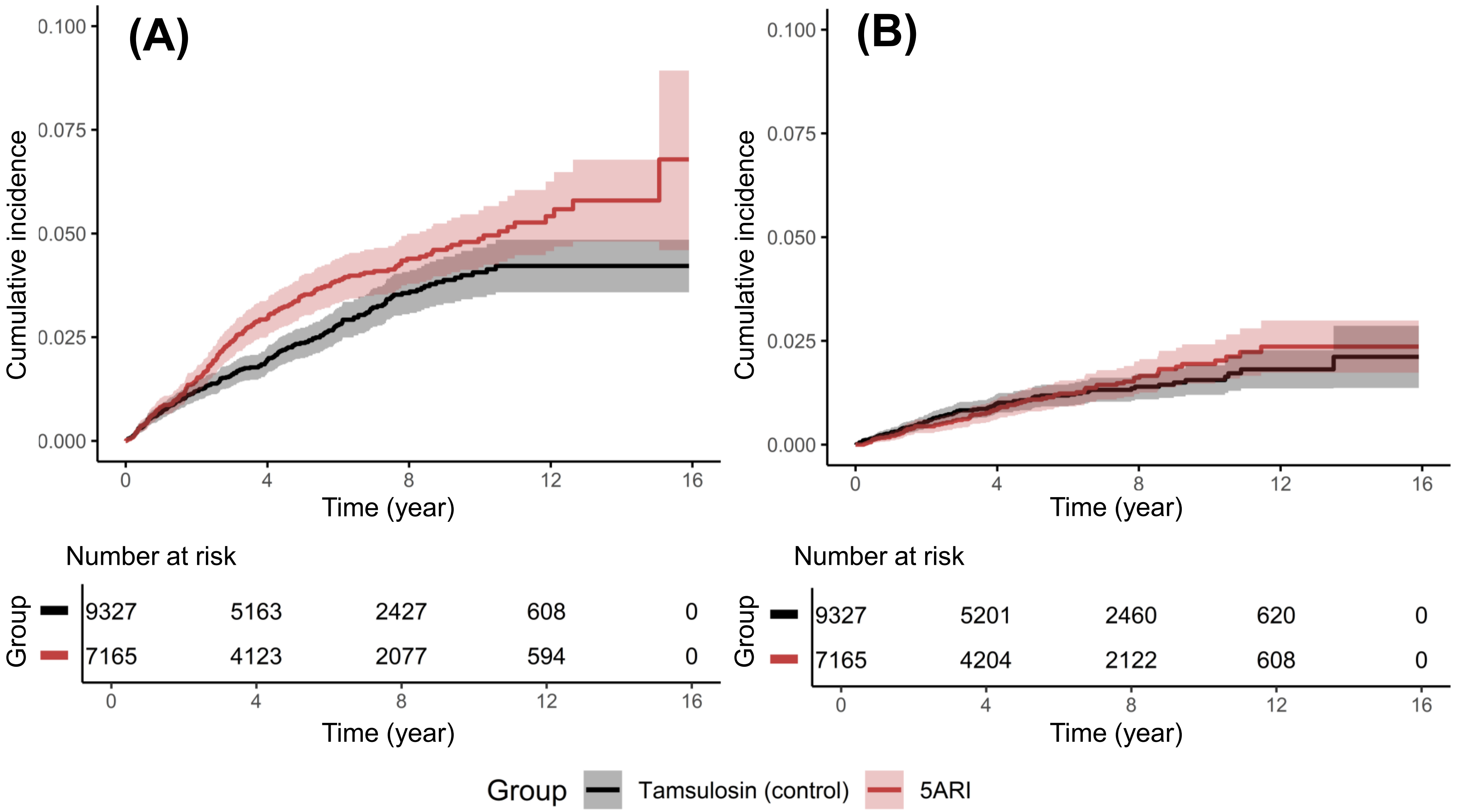

Tu et al. Supplementary Figure 2: Cumulative incidence plot of (A) myocardial infarction and (B) stroke in the matched diabetic cohort in IMRD-UK.

(A) SDRN-NDS

Outcome	Unadjusted HR	Adjusted HR
MACE	1.24 (1.11-1.38)	1.14 (1.01-1.28)
MI	1.25 (1.08-1.44)	1.19 (1.02-1.39)
Stroke	1.31 (1.09-1.59)	1.23 (1.01-1.51)
Cardiovascular death	1.11 (0.87-1.41)	0.93 (0.71-1.21)
Peripheral vascular diseases	1.21 (1.02-1.43)	1.25 (1.04-1.49)
Diabetic nephropathy	1.18 (0.95-1.46)	1.13 (0.90-1.42)
Background diabetic eye disease	1.00 (0.87-1.16)	1.13 (0.90-1.42)
Referrable diabetic eye disease	1.06 (0.90-1.25)	1.06 (0.89-1.26)
Diabetic neuropathy	1.10 (0.99-1.23)	1.04 (0.93-1.17)
Receipt of insulin	0.86 (0.76-0.98)	0.89 (0.77-1.01)

(B) IMRD-UK

Outcome	Unadjusted HR	Adjusted HR
MACE	1.21 (1.02-1.43)	1.14 (0.97-1.33)
MI	1.26 (1.03-1.53)	1.21 (1.00-1.45)
Stroke	1.10 (0.80-1.51)	1.00 (0.74-1.34)
Receipt of insulin	0.92 (0.81-1.05)	0.99 (0.86-1.14)

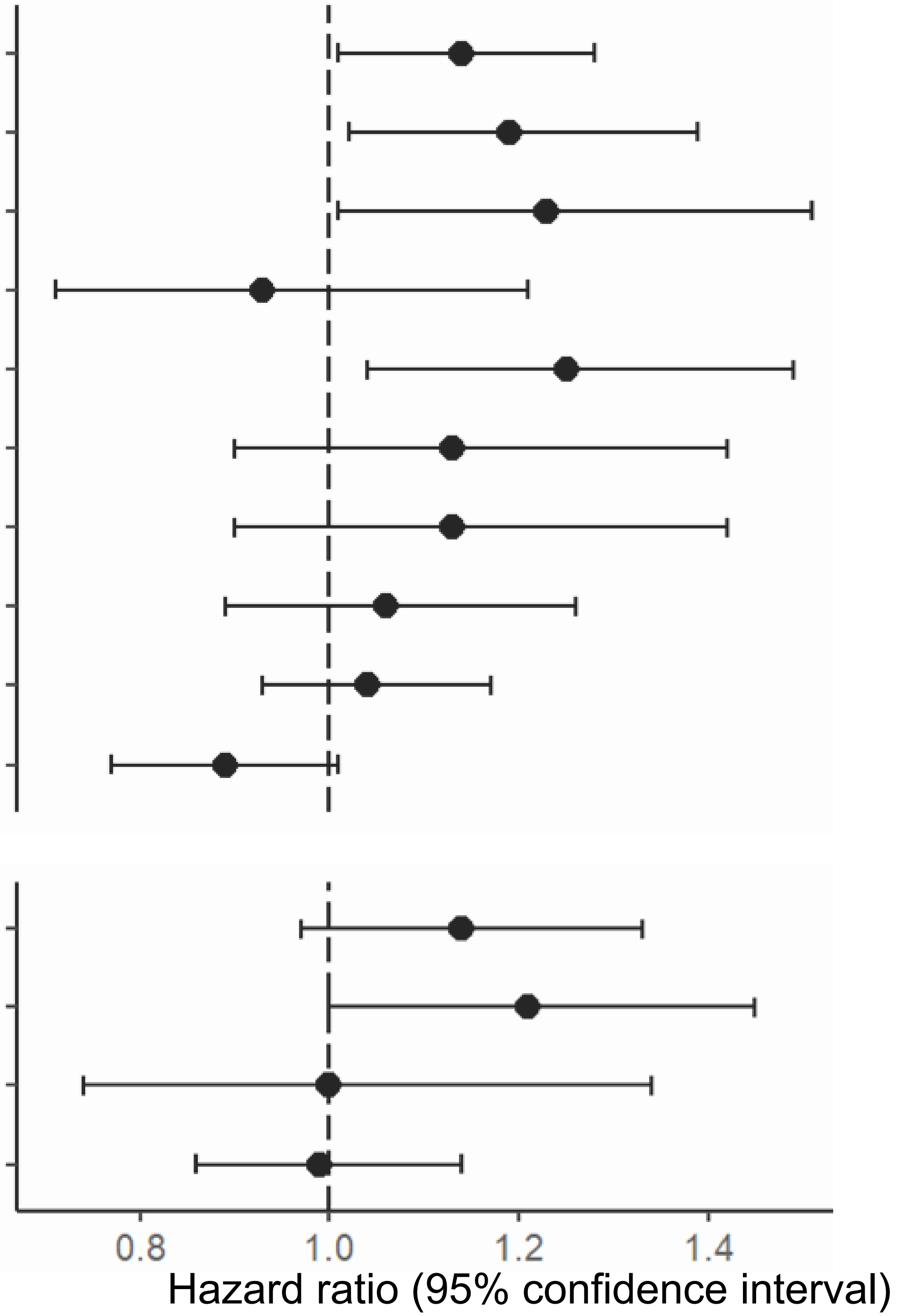

Tu et al. Supplementary Figure 3. Hazard ratio and 95% confidence interval (CI) for all outcomes of finasteride in comparison with tamsulosin in (A) SDRN-NDS and (B) diabetic cohort in IMRD-UK.

Abbreviations: MACE, major adverse cardiovascular events; MI, myocardial infarction.

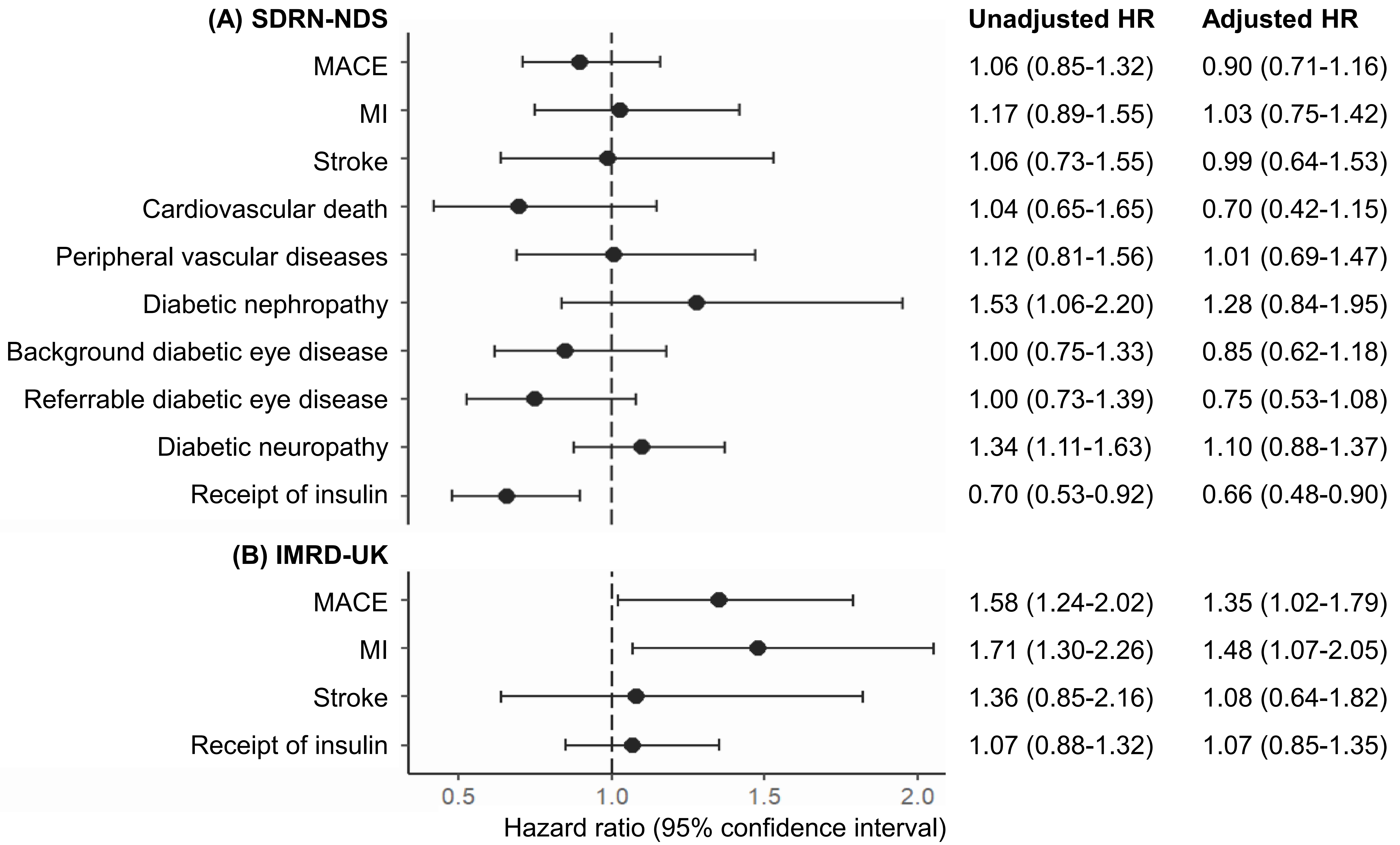

Tu et al. Supplementary Figure 4. Hazard ratio and 95% confidence interval (CI) for all outcomes of dutasteride in comparison with tamsulosin in (A) SDRN-NDS and (B) diabetic cohort in IMRD-UK.

Abbreviations: MACE, major adverse cardiovascular events; MI, myocardial infarction.

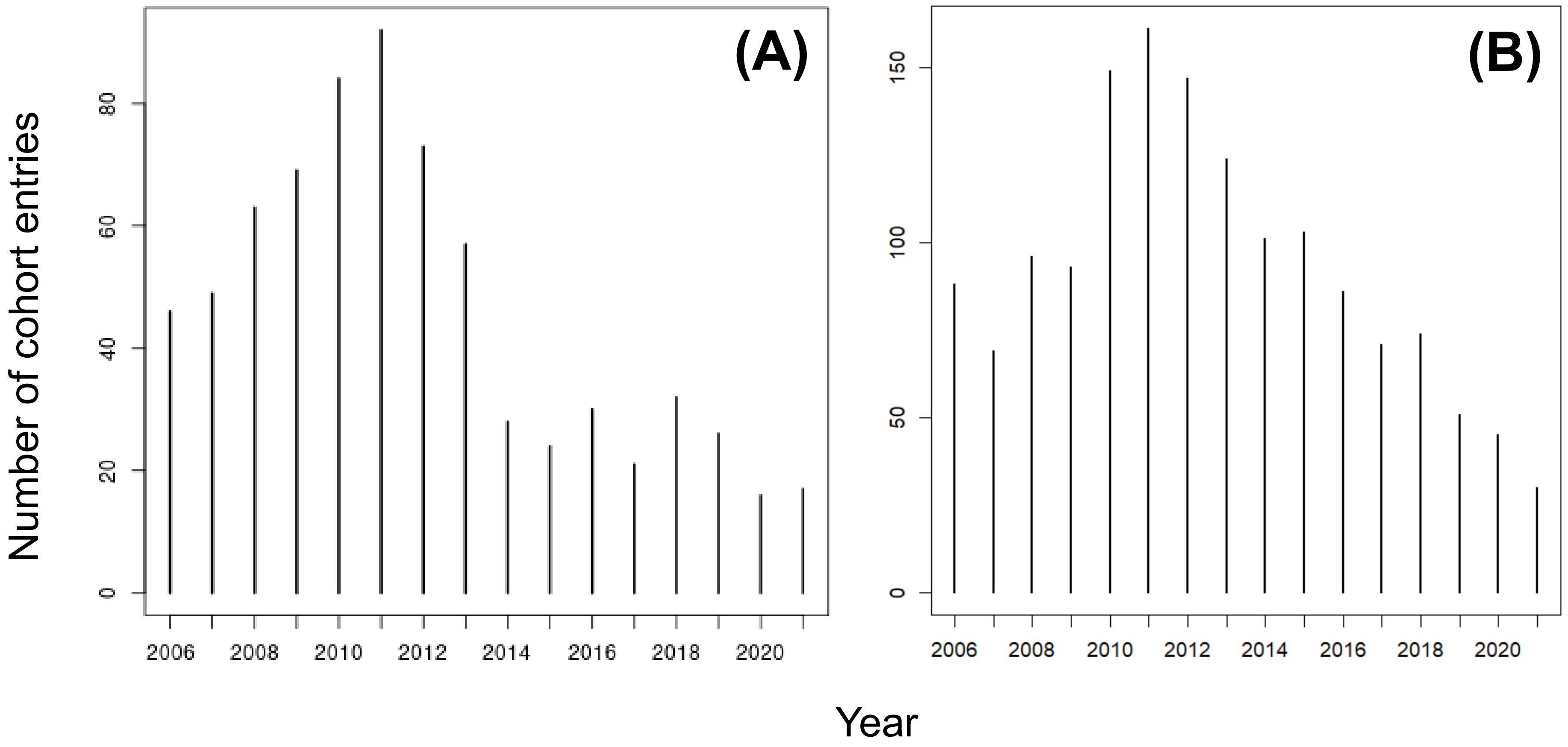

Supplementary Figure 5: Distribution of index dates in dutasteride groups in (A) SDRN-NDS and (B) diabetic cohort in IMRD-UK.
