# Supplementary material for: Cardiovascular safety of 5α-reductase inhibitors in people with benign prostatic hyperplasia and type 2 diabetes: a propensity score-matched analysis
Source: Eur Heart J Cardiovasc Pharmacother. 2026 Jan 17;12(2):97–107. doi: 10.1093/ehjcvp/pvag003 (PMC12946974; doi:10.1093/ehjcvp/pvag003)
Supplement: pvag003_Supplementary_Data [file pvag003_supplementary_data.zip › Tu2026.ehjcvp.supp_tables.pdf]

Tu et al. Supplementary Tables

Supplementary Table 1. Baseline characteristics of 5α-reductase inhibitors and tamsulosin groups before propensity score matching (SDRN-NDS)

	Tamsulosin	5ARI	SMD
**N**	6347	8340	
**Age**	70.5 (9.9)	73.9 (8.7)	0.359
**Diabetes duration (year)**	10.0 (7.5)	10.4 (7.5)	0.055
**SIMD quintile**			0.072
**1 (most deprived)**	1011 (15.9)	1500 (18.0)	
**2**	1396 (22.0)	1743 (20.9)	
**3**	1351 (21.3)	1797 (21.5)	
**4**	1332 (21.0)	1603 (19.2)	
**5 (least deprived)**	1009 (15.9)	1392 (16.7)	
**Missing**	248 (3.9)	305 (3.7)	
**BMI categories**			0.079
**< 25**	871 (13.7)	1303 (15.6)	
**25-30**	2287 (36.0)	3130 (37.5)	
**> 30**	3047 (48.0)	3699 (44.4)	
**Missing**	142 (2.2)	208 (2.5)	
**Smoking**			0.034
**No**	2322 (36.6)	3053 (36.6)	
**Former**	3005 (47.3)	4030 (48.3)	
**Yes**	789 (12.4)	996 (11.9)	
**Missing**	231 (3.6)	261 (3.1)	
**Calendar year**			0.158
**2006**	142 (2.2)	214 (2.6)	
**2007**	123 (1.9)	292 (3.5)	
**2008**	184 (2.9)	282 (3.4)	
**2009**	214 (3.4)	344 (4.1)	
**2010**	351 (5.5)	442 (5.3)	
**2011**	329 (5.2)	547 (6.6)	
**2012**	409 (6.4)	610 (7.3)	
**2013**	437 (6.9)	541 (6.5)	
**2014**	474 (7.5)	599 (7.2)	
**2015**	466 (7.3)	612 (7.3)	
**2016**	530 (8.4)	611 (7.3)	
**2017**	497 (7.8)	625 (7.5)	
**2018**	575 (9.1)	764 (9.2)	
**2019**	526 (8.3)	674 (8.1)	
**2020**	499 (7.9)	591 (7.1)	
**2021**	591 (9.3)	592 (7.1)	
**Ethnicity**			0.080
**White**	6468 (77.6)	4774 (75.2)	
**South Asian**	158 (1.9)	99 (1.6)	
**Black**	13 (0.2)	21 (0.3)	
**Chinese**	16 (0.2)	12 (0.2)	
**Other**	222 (2.7)	159 (2.5)	
**Unknown**	1463 (17.5)	1282 (20.2)	
**COPD**	462 (7.3)	720 (8.6)	0.050

**Cancer**	715 (11.3)	1017 (12.2)	0.029
**Biochemistry**			
**Total cholesterol (mmol/L)**	4.1 (0.9)	4.0 (0.85)	0.115
**Non-HDL (mmol/L)**	3.0 (0.9)	2.9 (0.85)	0.128
**Mean arterial pressure (mm Hg)**	95.7 (8.1)	94.7 (7.97)	0.132
**HbA_{1c} (mmol/mol)**	58.8 (17.2)	56.4 (15.1)	0.150
**Medication**			
**ACE inhibitors**	3132 (49.3)	4139 (49.6)	0.006
**ARB**	1119 (17.6)	1575 (18.9)	0.032
**Beta-blockers**	2120 (33.4)	2999 (36.0)	0.054
**Calcium channel blockers**	2330 (36.7)	3209 (38.5)	0.036
**Diuretics**	2142 (33.7)	3141 (37.7)	0.082
**Statins**	4965 (78.2)	6626 (79.4)	0.030
**Oral glucocorticoids**	905 (14.3)	1180 (14.1)	0.037
**NSAID**	1798 (28.3)	2478 (29.7)	0.030
**Glucose-lowering medications**	4701 (74.1)	6014 (72.1)	0.044
**Metformin**	3990 (62.9)	4913 (58.9)	0.081
**Sulfonylurea**	2037 (32.1)	2604 (31.2)	0.019
**DPP4 inhibitor**	678 (10.7)	811 (9.7)	0.032
**SGLT2 inhibitor**	378 (6.0)	319 (3.8)	0.099
**GLP1 RA**	202 (3.2)	195 (2.3)	0.052
**Number of glucose-lowering medications**			0.104
**0**	1646 (25.9)	2326 (27.9)	
**1**	2098 (33.1)	2846 (34.1)	
**2**	1468 (23.1)	1937 (23.2)	
**3**	670 (10.6)	808 (9.7)	
**3+**	465 (7.3)	423 (5.1)	
**Number of drugs**	1.43 (1.25)	1.32 (1.18)	0.091

Data represented in N (%) or mean (SD). Standardized mean difference (SMD) calculated with reference to tamsulosin group. *Abbreviations: 5ARI, 5α-reductase inhibitors; SIMD, Scottish index of multiple deprivation; COPD, chronic obstructive pulmonary disease; ARB, angiotensin II receptor blocker; NSAID, non-steroidal anti-inflammatory drugs; DPP4, dipeptidyl peptidase-4; SGLT2, sodium-glucose co-transporter-2 inhibitors; GLP-1 RA, glucagon-like peptide-1 (GLP-1) receptor agonists.*

Supplementary Table 2. Baseline characteristics with first-level matches under 1:2 variable ratio propensity score matching in SDRN-NDS cohort.

	Tamsulosin	5ARI	SMD
N	5768	5768	
Age	72.0 (8.9)	72.4 (8.8)	0.046
Diabetes duration (year)	10.2 (7.5)	10.2 (7.5)	0.002
SIMD quintile			0.029
1 (most deprived)	943 (16.3)	981 (17.0)	
2	1241 (21.5)	1200 (20.8)	
3	1238 (21.5)	1258 (21.8)	
4	1180 (20.5)	1158 (20.1)	
5 (least deprived)	941 (16.3)	960 (16.6)	
Missing	225 (3.9)	211 (3.7)	
BMI categories			0.031
< 25	805 (14.0)	842 (14.6)	
25-30	2118 (36.7)	2165 (37.5)	
> 30	2716 (47.1)	2629 (45.6)	
Missing	129 (2.2)	132 (2.3)	
Smoking			0.025
No	2087 (36.2)	2077 (36.0)	
Former	2787 (48.3)	2839 (49.2)	
Yes	706 (12.2)	664 (11.5)	
Missing	188 (3.3)	188 (3.3)	
COPD	455 (7.3)	474 (8.2)	0.033
Cancer	713 (11.5)	675 (11.7)	0.006
Biochemistry			
Total cholesterol (mmol/L)	4.1 (0.9)	4.1 (0.9)	0.001
Non-HDL (mmol/L)	2.9 (0.9)	2.9 (0.9)	0.003
Mean arterial pressure (mm Hg)	95.3 (7.9)	95.2 (8.0)	0.018
HbA_{1c} (mmol/mol)	58.5 (16.9)	56.8 (15.5)	0.093
Medication			
ACE inhibitors	2868 (49.7)	2852 (49.4)	0.006
ARB	1062 (18.4)	1041 (18.0)	0.009
Beta-blockers	2019 (35.0)	1987 (34.4)	0.012
Calcium channel blockers	2186 (37.9)	2174 (37.7)	0.004
Diuretics	2045 (35.5)	2042 (35.4)	0.001
Statins	4568 (79.2)	4552 (78.9)	0.007
Oral glucocorticoids	817 (14.2)	834 (14.5)	0.005
NSAID	1648 (28.6)	1657 (28.7)	0.003
Glucose-lowering medications	4236 (73.4)	4211 (73.0)	0.010
Metformin	3576 (62.0)	3481 (60.4)	0.034
Sulfonylurea	1856 (32.2)	1771 (30.7)	0.032
DPP4 inhibitor	597 (10.4)	589 (10.2)	0.005
SGLT2 inhibitor	300 (5.2)	251 (4.4)	0.040
GLP1 RA	166 (2.9)	154 (2.7)	0.013
Number of glucose-lowering medications			0.056
0	1532 (26.6)	1557 (27.0)	
1	1898 (32.9)	1977 (34.3)	

**2**	1344 (23.3)	1340 (23.2)	
**3**	611 (10.6)	577 (10.0)	
**3+**	383 (6.6)	317 (5.3)	
**Number of drugs**	1.40 (1.23)	1.35 (1.20)	0.043

Data represented in N (%) or mean (SD). Standardized mean difference (SMD) calculated with reference to tamsulosin group*. Abbreviations: 5ARI, 5α-reductase inhibitors; SIMD, Scottish index of multiple deprivation; COPD, chronic obstructive pulmonary disease; ARB, angiotensin II receptor blocker; NSAID, non-steroidal anti-inflammatory drugs; DPP4, dipeptidyl peptidase-4; SGLT2, sodium-glucose co-transporter-2 inhibitors; GLP-1 RA, glucagon-like peptide-1 (GLP-1) receptor agonists.*

Supplementary Table 3. Baseline characteristics of 5α-reductase inhibitors and tamsulosin groups before propensity score matching in the diabetic cohort in IMRD-UK

	Tamsulosin	5ARI	SMD
**N**	10038	7260	
**Age**	70.25 (9.95)	73.50 (8.77)	0.347
**GP visits in past year**	14.04 (10.32)	14.95 (10.31)	0.088
**Diabetes duration (year)**	8.59 (6.64)	9.05 (6.86)	0.068
**Townsend**			0.051
**1 (least deprived)**	1953 (19.5)	1394 (19.2)	
**2**	1880 (18.7)	1492 (20.6)	
**3**	1923 (19.2)	1345 (18.5)	
**4**	1682 (16.8)	1175 (16.2)	
**5 (most deprived)**	1165 (11.6)	794 (10.9)	
**Missing**	1435 (14.3)	1060 (14.6)	
**BMI category**			0.073
**<25**	1640 (16.3)	1252 (17.2)	
**25-30**	3789 (37.7)	2888 (39.8)	
**>30**	4273 (42.6)	2942 (40.5)	
**Missing**	336 (3.3)	178 (2.5)	
**Smoking**			0.087
**No**	5186 (51.7)	3915 (53.9)	
**Former**	6 (0.1)	8 (0.1)	
**Yes**	3643 (36.3)	2655 (36.6)	
**Missing**	1203 (12.0)	682 (9.4)	
**Alcohol consumption**			0.029
**No**	1627 (16.2)	1214 (16.7)	
**Former**	333 (3.3)	213 (2.9)	
**Yes**	896 (8.9)	616 (8.5)	
**Missing**	7182 (71.5)	5217 (71.9)	
**Calendar year**			0.117
**2006**	346 (3.4)	310 (4.3)	
**2007**	372 (3.7)	309 (4.3)	
**2008**	443 (4.4)	381 (5.2)	
**2009**	502 (5.0)	428 (5.9)	
**2010**	697 (6.9)	497 (6.8)	
**2011**	705 (7.0)	582 (8.0)	
**2012**	770 (7.7)	587 (8.1)	
**2013**	839 (8.4)	632 (8.7)	
**2014**	895 (8.9)	570 (7.9)	
**2015**	853 (8.5)	548 (7.5)	
**2016**	719 (7.2)	470 (6.5)	
**2017**	620 (6.2)	467 (6.4)	
**2018**	723 (7.2)	454 (6.3)	
**2019**	653 (6.5)	429 (5.9)	
**2020**	468 (4.7)	334 (4.6)	
**2021**	433 (4.3)	262 (3.6)	
**COPD**	1088 (10.8)	825 (11.4)	0.017

**Hypertension**	6403 (63.8)	4918 (67.7)	0.083
**Dyslipidaemia**	2214 (22.1)	1639 (22.6)	0.012
**Cancer**	2209 (22.0)	1793 (24.7)	0.064
**Baseline medications**			
**ACE inhibitors**	4628 (46.1)	3351 (46.2)	0.001
**ARB**	1678 (16.7)	1297 (17.9)	0.030
**Beta-blockers**	2609 (26.0)	2063 (28.4)	0.055
**Calcium channel blockers**	3425 (34.1)	2551 (35.1)	0.021
**Diuretics**	2543 (25.3)	2112 (29.1)	0.084
**Statins**	7372 (73.4)	5392 (74.3)	0.019
**Oral glucocorticoids**	550 (5.5)	395 (5.4)	0.002
**NSAID**	947 (9.4)	559 (7.7)	0.062
**Glucose-lowering medications**	7739 (77.1)	5355 (73.8)	0.078
**Metformin**	6915 (68.9)	4645 (64.0)	0.104
**Sulfonylurea**	3217 (32.0)	2213 (30.5)	0.034
**DPP4 inhibitor**	1246 (12.4)	834 (11.5)	0.029
**SGLT2 inhibitor**	387 (3.9)	204 (2.8)	0.058
**GLP-1 RA**	313 (3.1)	172 (2.4)	0.046
**Number of glucose-lowering medications**			0.094
**0**	2146 (21.4)	1789 (24.6)	
**1**	3908 (38.9)	2863 (39.4)	
**2**	2741 (27.3)	1829 (25.2)	
**3**	987 (9.8)	609 (8.4)	
**3+**	256 (2.6)	170 (2.3)	
**Number of drugs**	1.34 (1.01)	1.25 (1.00)	0.089

Data represented in N (%) or mean (SD). Standardized mean difference (SMD) calculated with reference to tamsulosin group. *Abbreviations: 5ARI, 5α-reductase inhibitors; COPD, chronic obstructive pulmonary disease; ARB, angiotensin II receptor blocker; NSAID, non-steroidal anti-inflammatory drugs; DPP4, dipeptidyl peptidase-4; SGLT2, sodium-glucose co-transporter-2 inhibitors; GLP-1 RA, glucagon-like peptide-1 (GLP-1) receptor agonists.*

Supplementary Table 4. Incidence rates per 10,000 person-years of all outcomes in (A) SDRN-NDS and (B) diabetic cohort in IMRD-UK.

	N of events/N of patients among matched 5ARI patients	N of events/N of patients among matched tamsulosin patients	Incidence among matched 5ARI patients (95% CI)	Incidence among matched tamsulosin patients (95% CI)
**(A) SDRN-NDS**				
**MACE**	573/5768	526/6201	212.0 (194.7,229.4)	191.8 (175.4,208.2)
**MI**	337/5768	297/6201	122.9 (109.8,136.0)	107.1 (94.9,119.3)
**Stroke**	197/5768	172/6201	71.0 (61.1,81.0)	61.2 (52.1,70.4)
**Cardiovascular death**	422/5768	419/6201	156.2 (141.3,171.1)	152.8 (138.2,167.4)
**Peripheral vascular diseases**	253/5356	222/5781	101.9 (89.3, 114.5)	86.7 (75.3, 98.2)
**Diabetic nephropathy**	155/5662	140/6131	59.2 (49.8, 68.5)	51.8 (43.2, 60.4)
**Background diabetic eye disease**	314/5166	315/5572	133.6 (118.8, 148.4)	131.1 (116.6, 145.6)
**Referrable diabetic eye diseases**	262/5274	249/5678	107.6 (94.5, 120.6)	101.2 (88.7, 113.8)
**Diabetic neuropathy**	577/4241	575/4627	308.6 (283.5, 333.8)	296.4 (272.2, 320.6)
**Receipt of insulin**	382/4998	454/5347	162.3 (146.0,178.6)	193.5 (175.7,211.3)
**(B) IMRD-UK**				
**MACE**	303/7165	306/9327	75.4 (66.9,83.9)	61.7 (54.8,68.6)
**MI**	232/7165	220/9327	57.4 (50.0,64.8)	44.0 (38.2,49.9)
**Stroke**	82/7165	95/9327	20.0 (15.7,24.3)	18.9 (15.1,22.7)
**Receipt of insulin**	242/6063	238/7940	74.4 (65.1,83.8)	58.1 (50.7,65.5)

Abbreviations: 5ARI, 5α-reductase inhibitors; MACE, major adverse cardiovascular event; MI, myocardial infarction.

Supplementary Table 5. Results of the sensitivity analyses in the SDRN-NDS cohort.

	Only group	Covid-19
**MACE**	1.18 [1.04-1.34]	1.01 [0.90-1.13]
**Non-fatal MI**	1.27 [1.08-1.50]	1.09 [0.94-1.26]
**Non-fatal stroke**	1.15 [0.92-1.44]	1.08 [0.89-1.30]
**Cardiovascular death**	0.98 [0.75-1.30]	0.80 [0.63-1.01]
**Peripheral vascular diseases**	1.27 [1.04-1.54]	1.10 [0.92-1.30]
**Diabetic nephropathy**	1.16 [0.91-1.49]	0.98 [0.79-1.22]
**Background diabetic eye disease**	1.11 [0.93-1.32]	1.03 [0.89-1.20]
**Referrable diabetic eye diseases**	1.16 [0.96-1.41]	0.98 [0.88-1.10]
**Diabetic neuropathy**	1.12 [0.98-1.27]	1.13 [1.02-1.26]
**Receipt of insulin**	0.87 [0.76-1.00]	0.84 [0.73-0.96]

Abbreviations: MACE, major adverse cardiovascular event; MI, myocardial infarction.

Supplementary Table 6. Results of the sensitivity analyses in the diabetic cohort in IMRD-UK.

	Only group	Covid-19
**MACE**	1.00 [0.85-1.17]	1.20 [1.02-1.41]
**MI**	1.11 [0.92-1.33]	1.27 [1.05-1.52]
**Stroke**	0.72 [0.53-0.96]	1.08 [0.81-1.46]
**Receipt of insulin**	1.06 [0.75-1.19]	1.00 [0.94-1.07]

Abbreviations: MACE, major adverse cardiovascular event; MI, myocardial infarction.

Supplementary Table 7. Results of the competing risk analyses.

	**SDRN-NDS**		**IMRD-UK (diabetic cohort)**	
	Cause-specific HR	Sub-distribution HR	Cause-specific HR	Sub-distribution HR
**MACE***	1.02 [0.91-1.14]	1.10 [0.99-1.23]	1.16 [0.99-1.36]	1.21 [1.03-1.41]
**MI**	1.10 [0.95-1.27]	1.15 [1.00-1.34]	1.25 [1.04-1.50]	1.29 [1.07-1.55]
**Stroke**	1.10 [0.91-1.33]	1.19 [0.98-1.43]	0.99 [0.74-1.33]	1.04 [0.77-1.40]
**Cardiovascular death**	0.78 [0.62-0.99]	0.86 [0.69-1.11]	/	/

*Major adverse cardiovascular event (MACE) was defined as non-fatal myocardial infarction (MI), non-fatal stroke, or cardiovascular death in SDRN-NDS, and fatal or non-fatal MI and stroke in IMRD-UK. The sub-distribution hazard ratio (HR) was computed using non-cardiovascular death as a competing event in SDRN-NDS and all-cause mortality in IMRD-UK. Cardiovascular death was not assessed in IMRD-UK as cause of death was not recorded.

Supplementary Table 8. Incidence rates per 10,000 person-years of all outcomes in the "Only" group sensitivity analysis for diabetic cohort in IMRD-UK.

**MACE**	61/1594	113/3174	66.4 (49.8,83.1)	65.4 (53.4,77.5)
**MI**	48/1594	80/3174	51.9 (37.2,66.5)	45.9 (35.8,56.0)
**Stroke**	14/1594	35/3174	14.9 (7.1,22.7)	19.9 (13.3,26.5)
**Receipt of insulin**	108/1350	226/2686	147.3 (119.5,175.1)	154.9 (134.7,175.1)

Abbreviations: 5ARI, 5α-reductase inhibitors; MACE, major adverse cardiovascular event; MI, myocardial infarction.

Supplementary Table 9. Baseline characteristics of 5α-reductase inhibitors and tamsulosin groups of all patients with BPH after 1:2 variable ratio propensity score matching (IMRD-UK).

	Tamsulosin	5ARI	SMD
**N**	58158	44809	
**Age**	69.22 (10.22)	71.63 (9.75)	0.242
**GP visits in past year**	10.58 (8.72)	11.44 (9.03)	0.096
**Townsend**			0.017
**1 (least deprived)**	14046 (24.2)	10768 (24.0)	
**2**	12403 (21.3)	9808 (21.9)	
**3**	10590 (18.2)	8175 (18.2)	
**4**	7987 (13.7)	5994 (13.4)	
**5 (most deprived)**	5222 (9.0)	3931 (8.8)	
**Missing**	7910 (13.6)	6133 (13.7)	
**BMI category**			0.039
**<25**	10964 (18.9)	8897 (19.9)	
**25-30**	17737 (30.5)	14062 (31.4)	
**>30**	12484 (21.5)	9139 (20.4)	
**Missing**	16973 (29.2)	12711 (28.4)	
**Smoking**			0.048
**No**	25535 (43.9)	19677 (43.9)	
**Former**	25525 (43.9)	20310 (45.3)	
**Yes**	6885 (11.8)	4662 (10.4)	
**Missing**	213 (0.4)	160 (0.4)	
**Alcohol consumption**			0.024
**No**	4185 (7.2)	3427 (7.6)	
**Former**	5724 (9.8)	4590 (10.2)	
**Yes**	44119 (75.9)	33730 (75.3)	
**Missing**	4130 (7.1)	3062 (6.8)	
**Calendar year**			0.089
**2006**	2652 (4.6)	2488 (5.6)	
**2007**	2909 (5.0)	2633 (5.9)	
**2008**	3249 (5.6)	2776 (6.2)	
**2009**	3470 (6.0)	3005 (6.7)	
**2010**	4327 (7.4)	3187 (7.1)	
**2011**	4374 (7.5)	3564 (8.0)	
**2012**	4727 (8.1)	3621 (8.1)	
**2013**	4977 (8.6)	3711 (8.3)	
**2014**	4989 (8.6)	3667 (8.2)	
**2015**	4323 (7.4)	3147 (7.0)	
**2016**	3674 (6.3)	2747 (6.1)	
**2017**	3286 (5.7)	2453 (5.5)	
**2018**	3504 (6.0)	2436 (5.4)	
**2019**	3231 (5.6)	2278 (5.1)	
**2020**	2431 (4.2)	1749 (3.9)	
**2021**	2035 (3.5)	1347 (3.0)	
**COPD**	5518 (9.5)	4487 (10.0)	0.018
**Hypertension**	25579 (44.0)	21045 (47.0)	0.060
**Dyslipidaemia**	9147 (15.7)	7262 (16.2)	0.013

**Type 2 diabetes**	9348 (16.1)	7124 (15.9)	0.005
**Cancer**	12385 (21.3)	10294 (23.0)	0.040
**Baseline medications**			
**ACE inhibitors**	13953 (24.0)	11286 (25.2)	0.028
**ARB**	5520 (9.5)	4554 (10.2)	0.023
**Beta-blockers**	9655 (16.6)	8273 (18.5)	0.049
**Calcium channel blockers**	13550 (23.3)	10767 (24.0)	0.017
**Diuretics**	9654 (16.6)	8201 (18.3)	0.045
**Statins**	23625 (40.6)	18777 (41.9)	0.026
**Oral glucocorticoids**	2848 (4.9)	2110 (4.7)	0.009
**NSAID**	6084 (10.5)	4176 (9.3)	0.038
**Glucose-lowering medications**	7218 (12.4)	5340 (11.9)	0.015
**Metformin**	6437 (11.1)	4636 (10.4)	0.023
**Sulfonylurea**	3013 (5.2)	2201 (4.9)	0.012
**DPP4 inhibitor**	1131 (1.9)	829 (1.9)	0.007
**SGLT2 inhibitor**	331 (0.6)	206 (0.5)	0.015
**GLP1 RA**	278 (0.5)	172 (0.4)	0.014

Data represented in N (%) or mean (SD). Standardized mean difference (SMD) calculated with reference to tamsulosin group. *Abbreviations: 5ARI, 5α-reductase inhibitors; GP, general practice; COPD, chronic obstructive pulmonary disease; ARB, angiotensin II receptor blocker; NSAID, non-steroidal anti-inflammatory drugs; DPP4, dipeptidyl peptidase-4; SGLT2, sodium-glucose co-transporter-2 inhibitors; GLP-1 RA, glucagon-like peptide-1 (GLP-1) receptor agonists.*

Supplementary Table 10. Hazard ratio and 95% confidence interval for finasteride and dutasteride in all BPH patients (IMRD-UK)

	Unadjusted HR	Adjusted HR
**MACE**		
**Finasteride vs Tamsulosin**	1.25 (1.16-1.35)	1.17 (1.09-1.26)
**Dutasteride vs Tamsulosin**	1.33 (1.19-1.49)	1.17 (1.02-1.33)
**Myocardial infarction**		
**Finasteride vs Tamsulosin**	1.26 (1.15-1.38)	1.19 (1.10-1.30)
**Dutasteride vs Tamsulosin**	1.41 (1.24-1.16)	1.23 (1.06-1.43)
**Stroke**		
**Finasteride vs Tamsulosin**	1.23 (1.07-1.41)	1.11 (0.98-1.27)
**Dutasteride vs Tamsulosin**	1.23 (0.98-1.51)	1.07 (0.84-1.36)
**Receipt of insulin**		
**Finasteride vs Tamsulosin**	0.92 (0.82-1.04)	0.92 (0.81-1.04)
**Dutasteride vs Tamsulosin**	1.02 (0.85-1.23)	1.05 (0.85-1.30)

Hazard ratio (HR) before (unadjusted) and after (adjusted) propensity score matching. Patients in the finasteride and dutasteride groups were matched to tamsulosin separately. *Abbreviations: MACE, major adverse cardiovascular events.*
